# Evaluation of two selection tests for recruitment into radiology specialty training

**DOI:** 10.1186/s12909-016-0687-0

**Published:** 2016-07-11

**Authors:** Fiona Patterson, Alec Knight, Liam McKnight, Thomas C. Booth

**Affiliations:** Department of Psychology, Downing Street, Cambridge, CB2 3 EB UK; Work Psychology Group, 27 Brunel Parkway, Pride Park, Derbyshire, DE24 8HR UK; Department of Radiology, Morriston Hospital, Swansea, West Glamorgan SA6 6NL UK; Department of Neuroradiology, King’s College Hospital NHS Foundation Trust, London, SE5 9RS UK

**Keywords:** Selection, Selecting, Shortlist, Shortlisting, Recruitment, Radiology, Secondary care, Specialty training

## Abstract

**Background:**

This study evaluated whether two selection tests previously validated for primary care General Practice (GP) trainee selection could provide a valid shortlisting selection method for entry into specialty training for the secondary care specialty of radiology.

**Methods:**

We conducted a retrospective analysis of data from radiology applicants who also applied to UK GP specialty training or Core Medical Training. The psychometric properties of the two selection tests, a clinical problem solving (CPS) test and situational judgement test (SJT), were analysed to evaluate their reliability. Predictive validity of the tests was analysed by comparing them with the current radiology selection assessments, and the licensure examination results taken after the first stage of training (Fellowship of the Royal College of Radiologists (FRCR) Part 1).

**Results:**

The internal reliability of the two selection tests in the radiology applicant sample was good (α ≥ 0.80). The average correlation with radiology shortlisting selection scores was *r* = 0.26 for the CPS (with *p* < 0.05 in 5 of 11 shortlisting centres), *r* = 0.15 for the SJT (with *p* < 0.05 in 2 of 11 shortlisting centres) and *r* = 0.25 (with *p* < 0.05 in 5 of 11 shortlisting centres) for the two tests combined. The CPS test scores significantly correlated with performance in both components of the FRCR Part 1 examinations (*r* = 0.5 anatomy; *r* = 0.4 physics; *p* < 0.05 for both). The SJT did not correlate with either component of the examination.

**Conclusions:**

The current CPS test may be an appropriate selection method for shortlisting in radiology but would benefit from further refinement for use in radiology to ensure that the test specification is relevant. The evidence on whether the SJT may be appropriate for shortlisting in radiology is limited. However, these results may be expected to some extent since the SJT is designed to measure non-academic attributes. Further validation work (e.g. with non-academic outcome variables) is required to evaluate whether an SJT will add value in recruitment for radiology specialty training and will further inform construct validity of SJTs as a selection methodology.

## Background

This paper describes an evaluation study exploring whether two shortlisting selection tests currently used for entry into training in primary care General Practice (GP) could provide a valid shortlisting selection method for recruitment into specialty training for the secondary care specialty of radiology. The aim of shortlisting is to reduce the number of candidates subsequently undergoing a structured, nearly hour long, radiology specialty training interview by a panel of radiologists.

To achieve a robust selection system, the most crucial step is to identify appropriate selection criteria [1]. Previous job analysis studies using a multi-source, multi-method approach, indicate that there are a common set of competency domains important across secondary care specialties, such as empathy, integrity and resilience. Therefore, it is plausible that selection tests used for selection into one specialty could be readily transferred for use on another speciality, however very little previous research has explored this proposition directly.

Practically, the use of machine-markable shortlisting selection tests could provide a standardised approach to enhance both the efficiency (i.e. reduced time and cost) [2, 3] and effectiveness (i.e. improved validity) [4–7] of the default shortlisting selection method where each candidate application form is analysed and assigned a score by a radiologist. This might go some way to addressing previous concerns of a chief medical officer for England stating that “Reform must take account of…weak selection and appointment procedures: these are not standardised and are frequently not informed by core competencies” [2]. There are no published studies exploring radiology shortlisting selection and the findings may be of particular interest to Health Departments and Radiology Faculties exploring centralised shortlisting in the UK (and Ireland, Singapore and Hong Kong where the Fellowship of the Royal College of Radiologists (FRCR) is examined three times a year) as well as elsewhere internationally.

The selection tests used for UK GP recruitment are: (1) a *clinical problem solving* (CPS) test, where candidates are presented with questions that require clinical knowledge to solve problems reflecting either a diagnostic process or a patient’s management strategy; and (2) a *situational judgement test* (SJT), where candidates are presented with work-related scenarios regarding professional dilemmas that they may encounter, and asked to judge the appropriateness of different potential responses. The SJT targets important non-academic attributes including integrity, empathy and ability to cope with pressure that have been identified as necessary for success in General Practice [5]. The tests used to select GP specialty trainees have shown good reliability and predictive validity [4, 5] and good reliability and validity in pilots for other medical specialties including Core Medical Training (CMT; a two year internal medicine programme prior to subspecialisation as a specialty trainee) [6] and the acute specialties [7]. Any new selection method must satisfy various psychometric and legal criteria including standardisation, reliability, validity and fairness [8–10].

A recent systematic review of selection systems for medical education shows there exists few longitudinal predictive validity studies of selection tests especially in postgraduate training [1]. Using a longitudinal design, in this study we explore the differential prediction of two selection tests, one focusing on clinical knowledge (CPS) and the other focusing on non-academic attributes (SJT). Clinical knowledge tests have been well established as good predictors of subsequent in-training and job performance [1]. However, relatively little is known about SJTs in predicting subsequent performance and theoretically, researchers have debated the construct validity of SJTs for selection purposes [4, 11, 12]. As such, depending on the outcomes of interest, one might expect differential prediction when comparing a clinical knowledge based selection test and an SJT, as both instruments purport to measure theoretically different constructs.

Specifically, this study evaluates the comparative reliability and validity, as well as item difficulty and quality of these two selection tests for selection into specialty training for radiology, specifically addressing the following three research questions:

1. *What is the internal reliability of the CPS and SJT selection tests for a radiology applicant sample?*
2. *What is the predictive validity of the CPS and SJT for performance on the Fellowship of the Royal College of Radiologists (FRCR) Part 1 examination (a knowledge-based licensure examination taken after the first stage of training)?*
3. *Are CPS and SJT items set at an appropriate level of difficulty, and of appropriate quality, for use with a radiology applicant sample?*

## Methods

### Sampling and assessments

The National Research Ethics Service provided confirmation that ethical approval was not necessary for this study. Selection data (including CPS and SJT scores and candidate demographics) were obtained from the GP National Recruitment Office for all applicants who applied for UK GP or CMT training in 2009. The CPS paper comprised 94 items, lasting 90 min; the SJT had 50 items, lasting 90 min. Example items are provided in Table 1. Scores on both tests were converted to a scale with a mean of 250 and a standard deviation of 40.

**Table 1:** Example items for the clinical problem solving and situational judgement tests

Example of CPS item	Example of SJT item
Reduced Vision	You are reviewing a routine drug chart for a patient with rheumatoid arthritis during an overnight shift. You notice that your consultant has inappropriately prescribed methotrexate 7.5 mg daily instead of weekly.
A. Basilar migraine	
B. Cerebral tumour	
C. Cranial arteritis	
D. Macular degeneration	
E. Central retinal artery occlusion	
	Rank in order the following actions in response to this situation (1 = Most appropriate; 5 = Least appropriate).
F. Central retinal vein occlusion	
G. Optic neuritis (demyelinating)	
H. Retinal detachment	
I. Tobacco optic neuropathy	
For each patient below select the SINGLE most likely diagnosis from the list above. Each option may be selected once, more than once or not at all.	A. Ask the nurses if the consultant has made any other drug errors recently
	B. Correct the prescription to 7.5 mg weekly
	C. Leave the prescription unchanged until the consultant ward round the following morning
1. A 75 year old man, who is a heavy smoker, with a blood pressure of 170/105, complains of floaters in the left eye for many months and flashing lights in bright sunlight. He has now noticed a “curtain” across his vision.	
	D. Phone the consultant at home to ask about changing the prescription
	E. Inform the patient of the error

A substantial proportion of radiology applicants also applied for GP training or CMT or both, and these individuals were the target sample for analysis. Anonymised radiology selection data were acquired from the UK Department of Health for all applicants applying to English radiology training schemes in 2009. Additionally, we acquired FRCR Part 1 examination scores from 2010. The physics component of the FRCR Part 1 examination consisted of true or false multiple-choice questions (MCQ) and was machine-marked. The anatomy component had 100 questions based on 20 electronic images. The answers were written and marked centrally by experienced examiners. Both components were criterion-referenced with standards set using the Anghoff method. These scores were compared to the available selection data.

### Reliability

Cronbach’s co-efficient alpha (α) is an index of reliability (internal consistency) of a test or scale, which is expressed as a number between 0 and 1 [13]. This was used to measure whether several items that propose to measure the same general construct produce similar scores. For selection tests in medicine, α ≥ 0.80 is considered to be acceptable [14].

### Item analysis

Item analysis was conducted to determine whether the difficulty and quality of each CPS and SJT item was appropriate [15]. *Item facility* (also known as item difficulty) is shown by the mean score for each item, representing the proportion of candidates answering the item correctly (e.g. mean of 0.60 corresponds to 60 % of candidates answering the item correctly). Items are classified into three categories of facility: *easy* ≥ 0.8; *moderate =* 0.6 < 0.80; *hard*: < 0.6. *Item quality* is determined by the correlation of the item with the overall test score, not including the item itself (i.e. the item’s partial correlation). This measure provides information about whether the item helps to distinguish between good and poor overall performers. Item quality was classified into three categories using item partial correlations: *good* ≥ 0.25; *moderate =* 0.18 < 0.25; *weak* < 0.18. As a test can be seen as a set of items that predict the test score, ideally, all items would have good correlations [14].

### Predictive validity

Pearson and Spearman correlations were performed for parametric and non-parametric data respectively. All statistical tests were performed using IBM SPSS statistical software. Student’s *t*-test was used to assess significance, which was set at *p* < 0.05 (although *p* < 0.01 was stated if this threshold was reached).

## Results

### Subject characteristics

**Fig. 1:**
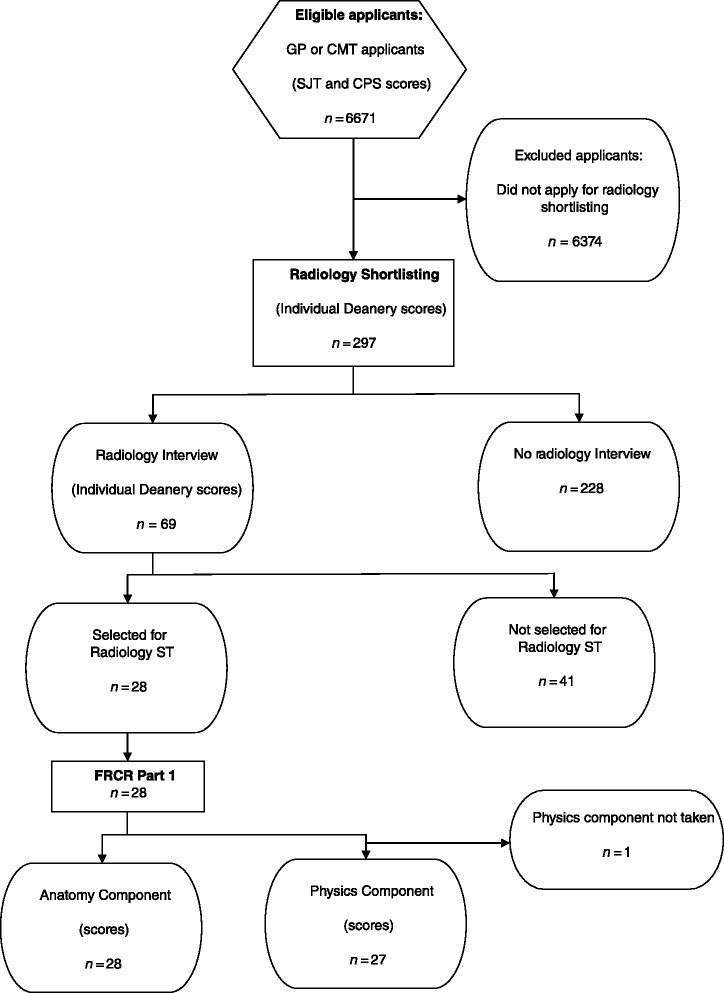
Flow diagram of radiology applicants who sat the Clinical Problem Solving and Situational Judgement Tests

Data for the combined GP/CMT population of 2009 applicants (*n* = 6671) were used for comparison with the radiology applicant sample (Fig. 1). Of 3108 radiology applications to 12 Deaneries (a Deanery is a regional organisation responsible for postgraduate medical training, within the structure of the UK National Health Service), 895 (28.8 %) of the applications were from individuals who had applied for GP training or CMT or both, and radiology shortlisting scores were available for 799 of the 895 (89.3 %) applications to 11 Deaneries. Because individuals could apply to more than one Deanery, this represented a total of 297 individual applicants. If an individual was successfully shortlisted they proceeded to interview. Of the cohort that had applied for GP training or CMT or both, radiology interview scores were available for a total of 69 shortlisting applications from 11 Deaneries. In 2009, both radiology shortlisting and radiology interviews were conducted by individual Deaneries rather than at a national level, so statistical correlations were conducted separately for each Deanery. Sample sizes from separate Deaneries were too small to conduct statistically meaningful correlations between the GP/CMT selection tests and radiology interview scores, but were of sufficient size to explore correlations between the GP/CMT selection tests and radiology shortlisting scores. The mean age of the radiology shortlisting sample was 31 years (range 24 – 46). Further demographic characteristics of the radiology shortlisting sample are presented in Table 2, showing a high proportion of Asian participants and participants from outside of the UK. The only available comparative demographic data of the radiology shortlisting cohort are those successfully recruited into radiology, which is a different subgroup. However, in contrast to all the GP/CMT applicants, the different radiology subgroups are similar in that the largest ethnic group is Asian and that there are more males than females.

**Table 2:** Demographic characteristics (a) The applicants to GP training and or CMT or both; (b) the applicants to GP training and or CMT or both who also underwent radiology shortlisting; and (c) those successfully recruited into radiology ST ^a^

		Shortlisting applicants		Recruited
		(a) GP/CMT	(b) Radiology	(c) Radiology
		(*n* = 6374)	(*n* = 297)	(*n* = 197)
		*n* (%)	*n* (%)	*n* (%)
Gender	Male	2806 (44.0)	168 (56.6)	126 (64.0)
	Female	3547 (55.6)	127 (42.7)	71 (36.0)
	Unreported	21 (0.3)	2 (0.7)	0 (0)
Ethnic Group	White British/Other	2914 (45.7)	60 (20.2)	84 (42.6)
	Asian	2464 (38.7)	186 (62.7)	89 (45.2)
	Other	996 (15.6)	51 (17.1)	24 (12.2)
Place of Medical Training	UK	3921 (61.5)	78 (26.3)	NK
	Non-UK	2453 (38.5)	219 (73.7)	NK

Abbreviation: *NK* not known

^a^Comparative radiology shortlisting demographic characteristics for the entire population had not been collated centrally in 2009. However, the RCR collated demographic data on those successfully recruited into radiology which are a different subgroup, but provide the best available comparator. There was no data on place of medical training

We obtained results for all radiology specialty trainees sitting the 2010 FRCR Part 1 examination (*n* = 1557). Twenty-eight and 27 of these sitting the anatomy and physics components respectively had also applied for GP training or CMT or both.

### Psychometric properties of CPS and SJT

In the radiology applicant sample, results showed that both the CPS and the SJT had good internal reliability (α = 0.80 and α = 0.84 respectively; Table 3).

**Table 3:** Clinical problem solving and situational judgement test descriptive statistics

	Clinical Problem Solving^a^		Situational Judgement Test^a^	
	Radiology sample (*n* = 297)	GP/CMT population (*n* = 6671)	Radiology sample (*n* = 297)	GP/CMT population (*n* = 6671)
Score	235.0	249.8	230.4	249.3
Mean (standard)				
Standard deviation	35.6	40.4	40.4	41.0
Range	91 - 315	56 - 342	58 - 312	50 -331
Reliability^b^ (α)	0.80	0.86	0.84	0.86

^a^Radiology sample and GP/CMT population compared gave CPS and SJT *p* < 0.001 (SJT *t* = 8.2, CPS *t* = 7.3; unpaired, 2-tailed *t* test)

^b^Using Cronbach’s co-efficient where α ≥ 0.8 is considered ‘good’ internal reliability

For the CPS, the mean item facility in the radiology sample was 0.72 (range 0.18 to 0.98). This moderate value was similar to the mean item facility in the GP/CMT population, which was 0.76 (range 0.26 to 0.97). There was deterioration in item quality in the radiology applicant sample, with 49 out of 94 (52 %) of items classified as weak compared with 18 out of 94 (19 %) in the GP/CMT population (Table 4).

**Table 4:** Item analysis (facility and quality) for clinical problem solving and situational judgement test. In addition to item analysis for the radiology shortlisting sample, item analysis for the GP/CMT population is also shown

	Clinical Problem Solving				Situational Judgement Test			
	Item facility *n* (*n*) ^a,b^				Item facility *n* (*n*) ^a,b^			
	Easy	Moderate	Hard	Total	Easy	Moderate	Hard	Total
Item quality^c^								
Good	11 (22)	10 (11)	5 (5)	26 (38)	5 (13)	16 (15)	3 (1)	24 (29)
Moderate	3 (19)	9 (12)	7 (7)	19 (38)	10 (11)	7 (4)	0 (1)	17 (16)
Weak	24 (8)	16 (8)	9 (2)	49 (18)	8 (4)	0 (1)	1 (0)	9 (5)
Total	38 (49)	35 (31)	21 (14)	94	23 (28)	23 (20)	4 (2)	50

^a^The data refer to item number for the radiology shortlisting sample and, in parentheses, the GP/CMT population

^b^Item facility is the proportion of candidates answering the item correctly (easy ≥ 0.8; moderate = 0.6 - 0.79; hard: < 0.6)

^c^Item quality is determined by the correlation of the item with the overall test score (good ≥ 0.25; moderate = 0.18 - 0.24; weak < 0.18)

For the SJT, the mean item facility in the radiology sample was 0.63 (range 0.16 to 0.91). This moderate value was also similar to the mean item facility in the GP/CMT population, which was 0.65 (range 0.18 to 0.92). There was slight deterioration in item quality in the radiology applicant sample, with 9 out of 50 (18 %) of items classified as weak compared with 5 out of 50 (10 %) in the GP/CMT population.

### CPS and SJT validity

We examined the predictive validity of the CPS test and the SJT by evaluating the extent to which tests scores correlated with (1) current radiology selection assessments used for shortlisting purposes, and (2) with subsequent performance in the FRCR Part 1 examination.

In 2009, shortlisting was conducted by individual Deaneries rather than at a national level, so analysis was performed for each Deanery separately (Table 5). Significant correlations were found between CPS and radiology shortlisting scores for candidates who applied to 5 of the 11 Deaneries. There were significant correlations for only 2 of the 11 Deaneries when SJT and radiology shortlisting scores were compared. The mean uncorrected correlation with radiology shortlisting scores was *r* = 0.26 for the CPS, *r* = 0.15 for the SJT and *r* = 0.25 for both GP selection assessments combined.

**Table 5:** Predictive validity of clinical problem solving and situational judgement tests in determining radiology shortlisting scores

Deanery	*n*	Score ^a^ (mean)	Score (SD)	CPS & shortlisting (*r*)	SJT & shortlisting (*r*)	Total & shortlisting (*r*)
East of England	79	16.7	6.9	0.23 ^b^	0.36 ^c^	0.36 ^c^
East Midlands	58	72.6	21.1	0.16	0.18	0.22
London	98	60.5	12.3	0.42 ^c^	0.45 ^c^	0.52 ^c^
Mersey	74	20.7	7.1	0.19	0.01	0.10
North Western	83	18.1	7.8	0.37 ^c^	0.20	0.34 ^c^
Oxford	68	12.9	3.0	0.20	0.16	0.22
Peninsula	54	31.3	10.7	0.15	0.00	0.10
Severn	53	54.8	10.8	0.53 ^c^	0.13	0.42 ^c^
Wessex	26	86.6	6	0.24	0.06	0.21
West Midlands	81	13.3	7.3	0.11	0.02	0.08
Yorks & Humber	125	10.5	3.5	0.25 ^c^	0.06	0.19 ^b^
Total significant				5	2	5
*p* < 0.05 (*n*)						

^a^ Each deanery had their own personal specification, scoring criteria and threshold score required for interviews across the country. Raw scores shown

^b^ *p* < 0.05; ^c^ *p* < 0.01 (*t*-test, 2-tailed)

The FRCR part 1 examination results were categorical (pass or fail). Therefore, non-parametric Spearman correlation coefficients were calculated for the two GP selection assessments and examination performance (Table 6). There was a significant correlation between the CPS scores and performance in both the anatomy (*r* = 0.50, *p* < 0.01) and physics (*r* = 0.42, *p* < 0.05) components. The SJT did not significantly correlate with either component of the FRCR Part 1 examination (*r* = -0.08 for anatomy; *r* = -0.02 for physics).

**Table 6:** Predictive validity of CPS and SJT in determining subsequent anatomy and physics examination results

	Examination	
	Anatomy (*n* = 28) (*r*)	Physics (*n* = 27) (*r*)
Selection test SJT	-0.08	-0.02
CPS	0.50 ^b^	0.42 ^a^
CPS & SJT combined score	0.46 ^a^	0.28

^a^ *p* < 0.05; ^b^ *p* < 0.01 (*t*-test, 2-tailed)

## Discussion

### Summary of findings

The CPS and the SJT both have acceptable internal reliability when used in a sample of candidates who subsequently underwent radiology shortlisting. In the same sample, the item facility was satisfactory for both CPS and SJT, although the item quality was unsatisfactory for the CPS test. The predictive validity analysis of the CPS test and the SJT suggests that, in the current formats, the CPS test had more predictive validity in radiology shortlisting than the SJT. The predictive validity analysis of the CPS test and the SJT demonstrated that the CPS correlated well with both components of the FRCR Part 1 examination while the SJT did not.

### Strengths and weaknesses of the study

Our inferences on CPS test and SJT psychometric properties for those applying to radiology specialty training are likely to be accurate as 100 % of the data were acquired. As almost 90 % of the radiology shortlisting data and 100 % of 2010 FRCR Part 1 examination data were captured from those who had undergone GP/CMT selection, our inferences on predictive validity are likely to be representative for this sample. Nonetheless this study has limitations. Because the necessary inclusion criterion consisted of radiologists who had undertaken the CPS test and SJT, the sample was inevitably a subset of the entire 2009 radiology applicant population, the majority of whom did not apply for GP training or CMT. Therefore, although indirect evidence suggested that the demographic characteristics were broadly representative of 2009 radiology applicants, there may have been sampling bias. For example, some of these trainees might have been unsuccessful at GP training/CMT selection and therefore radiology may have been a second career choice. A similar limitation is that applicants to radiology specialty training who were successfully recruited into radiology specialty training were inevitably a much smaller group than those undergoing shortlisting and a smaller group than those attending interview. Therefore, the numbers of those sitting the 2010 FRCR Part 1 examination were also small and subject to sampling bias.

As shortlisting is a means to select a small number of applicants for interview, few participants who underwent radiology shortlisting were subsequently interviewed for radiology specialty training. Since radiology interviews were implemented in 11 regions in 2009, the small interviewee sample sizes from each Deanery did not permit meaningful statistical analysis. Despite this being part of our aim, we were unable to explore meaningfully this third outcome measure. Outcome measures used were, therefore, the scores from radiology shortlisting and the first examination taken by the successful radiology trainees after the first stage of training (FRCR Part 1). Scores from a radiological examination seemed a suitable outcome to assess, and it is noted that similar predictive validity analysis was used in GP selection where future performance in the MRCGP was predicted [16]. Although an imperfect assessment of subsequent candidate ‘success’, these were the best data that could be obtained. Indeed, these were the only outcomes where candidates sat the same assessment that followed the CPS test and SJT. By the time the cohort sat the FRCR 2A or 2B examinations (licensure examinations taken after the later stages of training), the group was split in terms of both different examination sittings and the number of modules taken at any one time confounding statistical analysis. Furthermore, other qualitative outcomes, such as the Record of In-Training Assessment (RITA) or Annual Review of Competence Progression (ARCP) have been in different states of evolution since 2009 and were performed by different Deaneries, rendering the sample sizes too small for meaningful statistical analysis.

### Study explanations and recommendations

This study allowed us to explore whether selection tests that already exist for selection into other medical specialties could be translated to radiology specialty training selection. In terms of operational validity and candidate acceptance, the combination of the current CPS and SJT has proved to be the most effective in predicting selection outcomes when a batch of several tests was evaluated for GP training selection in 2009 [12], therefore it was plausible that the current CPS and SJT would predict selection outcomes in radiology specialty training selection. Knowing whether the current CPS and SJT currently used for GP and CMT selection were valid tests for radiology specialty training selection would determine whether there was a possibility to roll out these well-researched standardised tests to all radiology applicants which would likely enhance both the efficiency (i.e. reduced time, effort and cost) and effectiveness (i.e. test validity) of radiology selection. After all, since 2012 there has been no shortlisting and all eligible radiology specialty training candidates are now interviewed, with considerable cost and logistic implications. Therefore, the findings may be of particular interest to Health Departments and Radiology Faculties exploring centralised shortlisting in the UK (and also internationally including Ireland, Singapore and Hong Kong where the Fellowship of the Royal College of Radiologists (FRCR) is examined three times a year) as well as elsewhere around the globe.

The CPS and the SJT both have acceptable internal reliability when used in a sample of candidates who subsequently underwent radiology shortlisting. This shows that the previously published acceptable internal reliability for these tests [5] is also acceptable when a subset is analysed that applies to radiology. However, we found that the difficulty and quality of some of the items in the CPS test, and to a lesser extent the SJT, may be less appropriate for selection into radiology compared to GP training or CMT. Nonetheless, the CPS test scores appeared to be predictive of performance in radiology shortlisting in 5 of 11 Deaneries, and both FRCR Part 1 examinations. This supports the notion that there is measurable overlap in the constructs targeted by the CPS test and these radiology-specific assessments. Although radiology is a diagnostic-based specialty, these results replicate findings reported elsewhere for clinical-based specialties [6, 7]. Further work into radiology-specific CPS test items is needed to improve item quality, which may improve predictive validity in radiology shortlisting.

There were no significant correlations between the SJT and performance in either of the FRCR Part 1 examinations. The absence of significant correlations between these tests and the SJT might be explained by the fact that the tests are assessing different constructs: the SJT is designed to assess non-academic attributes such as integrity and coping with pressure, while the anatomy and physics examinations assess learned declarative knowledge in those areas. The SJT appeared to be predictive of performance in radiology shortlisting in 2 of the 11 Deaneries and the size of the validity coefficients varied considerably (with *r* ranging from 0.0 to 0.45). Therefore, there appears to be little overlap in the constructs targeted by the SJT and these radiology-specific assessments. Future research should explore outcome measures that relate to important non-academic attributes in order to judge the quality of the SJT for selection purposes.

When comparing the two selection tests, this study offers further support to exploring the construct validity of SJTs in particular, which has been a topic of considerable debate [1]. Our study supports the notion that SJTs are not measuring knowledge per se, but are measuring non-academic attributes. Theoretically, SJTs are thought to measure *prosocial implicit trait policies* which are an individual’s beliefs about the cost/benefits or effectiveness of different behaviours in particular situations. For example, a doctor dealing with a sensitive situation in the workplace (such as the death of a relative) may have to make a judgement that the situation demands an expression of empathy and agreeableness as a more successful strategy than lacking empathy or being disagreeable (even if the doctor is generally disagreeable or lacks empathy themselves). Given that there was no correlation observed between the SJT and subsequent performance in a clinical knowledge exam, this differential finding might be argued by some to support the construct validity of the SJT.

This has important implications for further international research in using SJTs for postgraduate selection. There exists little current research relating to the use of SJTs in selection in other contexts around the globe. Further research could explore the extent to which SJTs are relevant to selection in other countries and international job analysis studies may uncover the need to focus on different non-academic attributes depending on the local health system [17].

It is noticeable that there is heterogeneity in the data between Deaneries. For example, radiology shortlisting scores in London and the East of England Deaneries correlated well with the two GP selection assessments whereas radiology shortlisting scores in the Mersey, Peninsula and West Midlands Deaneries correlated poorly with the two GP selection assessments. Although not the focus of this paper, it is possible that the Deanery-specific radiology shortlisting method used in some Deaneries was more valid than the method used in other Deaneries.

Selection methods such as those used in GP selection provide a standardised shortlisting selection process that is likely to increase utility substantially once the initial development phase has been completed [11, 18]. The GP selection tests are completed under invigilated conditions and are machine-marked; therefore, they have significant advantages over the use of other (relatively unstandardised) approaches and could provide a cost-effective, standardised approach. Based on the evidence available, findings indicate that with further refinement, CPS tests may be appropriate assessments for selection in radiology. However, in order to use GP (or similar) SJT assessments in radiology selection, supportive evidence on validity is required. Further steps to develop both tests must be considered in the first instance.

Previous job analysis studies [19] suggests that different specialties place greater priority on certain competency domains that reflect the nature of the job role. Here, future research could explore the differences between the most important selection criteria for radiology compared to general practice.

First, it is recommended that a specific job analysis be conducted for radiology to ensure that all selection methods are targeting appropriate criteria. An initial job analysis was conducted for the GP specialty training role prior to the development of the GP selection assessments, [17] which was essential to ensure the content validity of the selection process (i.e. the degree to which individual test components represent GP-orientated clinical problem-solving and professional attributes targeted by the CPS and SJT respectively). Evidence sought through a job analysis study would further inform relevant stakeholders regarding the development of a selection assessment specification for radiology [14].

Second, a test specification for the CPS and SJT would need to be developed and agreed by key stakeholders in the radiology community to ensure that item content of any operational test is relevant and appropriate for radiology. These measures are likely to improve the item quality demonstrated in this study.

Third, once a radiology-specific CPS test and SJT are developed, further analyses should be conducted to determine the predictive validity of the assessments using larger samples that are likely to be more representative of the radiology applicant population as a whole. Furthermore, comparisons of radiology interview scores and the CPS and SJT scores would become possible and provide useful supplementary information.

## Conclusions

This research is an exploratory study examining the viability of the CPS test and SJT that are currently used for shortlisting of candidates for GP training, for use in radiology specialty training shortlisting selection. Findings indicate that with further refinement, although initially designed for selection into primary care, the CPS test may be a valid assessment for shortlisting in radiology specialty training and potentially other secondary care specialties. As might be hypothesised, the SJT did not correlate with knowledge-based outcomes as the criterion. However, further evaluations with different outcome variables that are related to important non-academic attributes (e.g. empathy, integrity, teamwork) are an important avenue for future research and is likely to enhance evidence for construct validity. We have made recommendations for future development of a radiology-specific CPS test and SJT that parallel the steps taken prior to the implementation of these selection tests for GP training shortlisting. With appropriate design, previous research shows that SJTs can add significant value in selection processes [20] and especially for recruitment into medicine [21]. In addition, development of alternative or additive radiology-specific selection tests aimed at diagnostics or visual perception might also be worthy areas for future research. Finally, the development of a multispecialty specialty training shortlisting selection test may be another direction for future research that would offer multiplicative efficiency savings.

## Abbreviations

ARCP, Annual Review of Competence Progression; CMT, core medical training; CPS, clinical problem solving test; FRCR, Fellowship of the Royal College of Radiologists; GP, general practice; MCQ, multiple-choice questions; MRCGP, Membership of the Royal College of General Paractitioners; RITA, record of in-training assessment; SJT, situational judgement test; α, Cronbach’s co-efficient alpha.
